# Infective Endocarditis Complicated by Iatrogenic Intracranial Hemorrhage Secondary to Cefazolin-Induced Coagulopathy

**DOI:** 10.7759/cureus.44875

**Published:** 2023-09-07

**Authors:** Siva Naga S Yarrarapu, Abdul-Rahaman Adedolapo Ottun, FNU Arty, Jayasree Ravilla, Gaurav Mohan, Muhammad Tayyeb, David Anwar

**Affiliations:** 1 Internal Medicine, Monmouth Medical Center, Long Branch, USA; 2 Internal Medicine, Presbyterian Hospital, Agogo, GHA; 3 Internal Medicine, University of Ghana, Accra, GHA; 4 Cardiology, Monmouth Medical Center, Long Branch, USA

**Keywords:** iatrogenic intracranial, elevated inr, infective endocarditis, cefazolin, prosthetic valve infective endocarditis

## Abstract

Infective endocarditis can be acute or subacute. It can be caused by viral, bacterial, fungal, and sometimes nonbacterial etiologies. It is an important cause of mortality and morbidity in children as well as adolescents, despite advances in management. A 59-year-old male with a past medical history of aortic valve (AV) replacement on warfarin presented to the Emergency Department with dull right flank pain and poor dentition on examination. Computerized tomography (CT) scans of the abdomen revealed the presence of splenic and renal infarcts. Warfarin was held after the international normalized ratio (INR) was noted to be elevated at 11. Following the activation of the sepsis bundle in the ER, he received intravenous fluids (30 cc/kg) and was started on vancomycin and ceftriaxone. On further evaluation, the transesophageal echocardiogram revealed mobile densities on the aortic surface concerning vegetation. Antibiotics were transitioned to cefazolin, gentamycin, and rifampin for the management of prosthetic valve endocarditis. The patient's INR improved to 3.5 on the third day of hospitalization, and heparin was initiated to maintain anticoagulation for the prosthetic valve. However, on the eighth day of hospitalization, the patient developed left-sided weakness and slurred speech. The CT head showed acute frontoparietal intracranial hemorrhage (ICH), with an INR noted to be 5. Heparin was reversed with protamine sulfate, and vitamin K was administered, following which the INR improved to 2.3. The patient was transferred to intensive care, but on the second day of the ICU stay, the INR again shot up to 6 with normal LFTS. The patient received vitamin K, but the INR only improved to 5. Subsequently, antibiotics were changed from cefazolin to nafcillin. INR thus fell to 1.6 in two days after changing the antibiotics. The patient was soon transferred to a higher center for aortic valve replacement. While few case reports have described severe coagulopathy induced by cefazolin, it is particularly seen with impaired renal function; however, our patient's renal function was completely normal. Coagulopathy is due to the drug's effect on intestinal flora and its structural methyl-thiadiazole side chain, which has similar effects as epoxide reductase inhibitors and results in INR elevation. Patients on cefazolin need to be closely monitored for INR levels every day, as there is a high likelihood of developing complications like ICH, as noted in this patient. While the monitoring of cefazolin levels is not necessarily indicated, it is necessary to place patients on fall precautions and monitor INR levels every day, as mentioned above.

## Introduction

Infective endocarditis (IE) can be acute or subacute. It can be caused by viral, bacterial, fungal, and nonbacterial etiologies. The most common organisms are *Staphylococcus aureus* and *Streptococcus viridians*. IE is diagnosed by a combination of clinical features, blood cultures, and positive echocardiogram findings [1,2]. Despite advances in management, the mortality rate with IE is relatively high, standing at 15%-30%. IE is usually managed with antibiotics for 4-6 weeks, but surgical intervention is required in about 50% of the cases, like patients with heart failure, prosthetic valve endocarditis, large vegetation, and persistent infections. It should be done during the same hospital admission. Early surgery within seven days of antibiotic therapy is associated with a lower risk of mortality in comparison to delayed surgery after 8-20 days of antibiotic treatment [3,4]. Antibiotics are selected based on culture results, but cefazolin is commonly used empirically as it covers gram-positive and gram-negative organisms, and the dose can be adjusted in renal disease easily. Cefazolin is associated with an adverse effect of coagulopathy, which is less known and rarely reported [5]. Herein, we present a case of infective endocarditis complicated by intracranial hemorrhage secondary to cefazolin use.

## Case presentation

**Figure 1:**
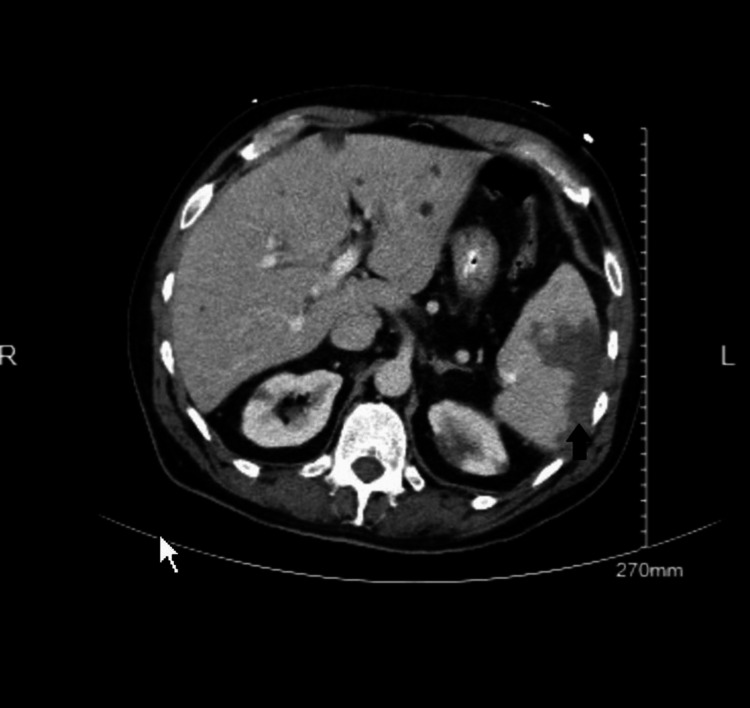
CT abdomen and pelvis demonstrate multiple wedge-shaped infarcts in the spleen.

**Figure 2:**
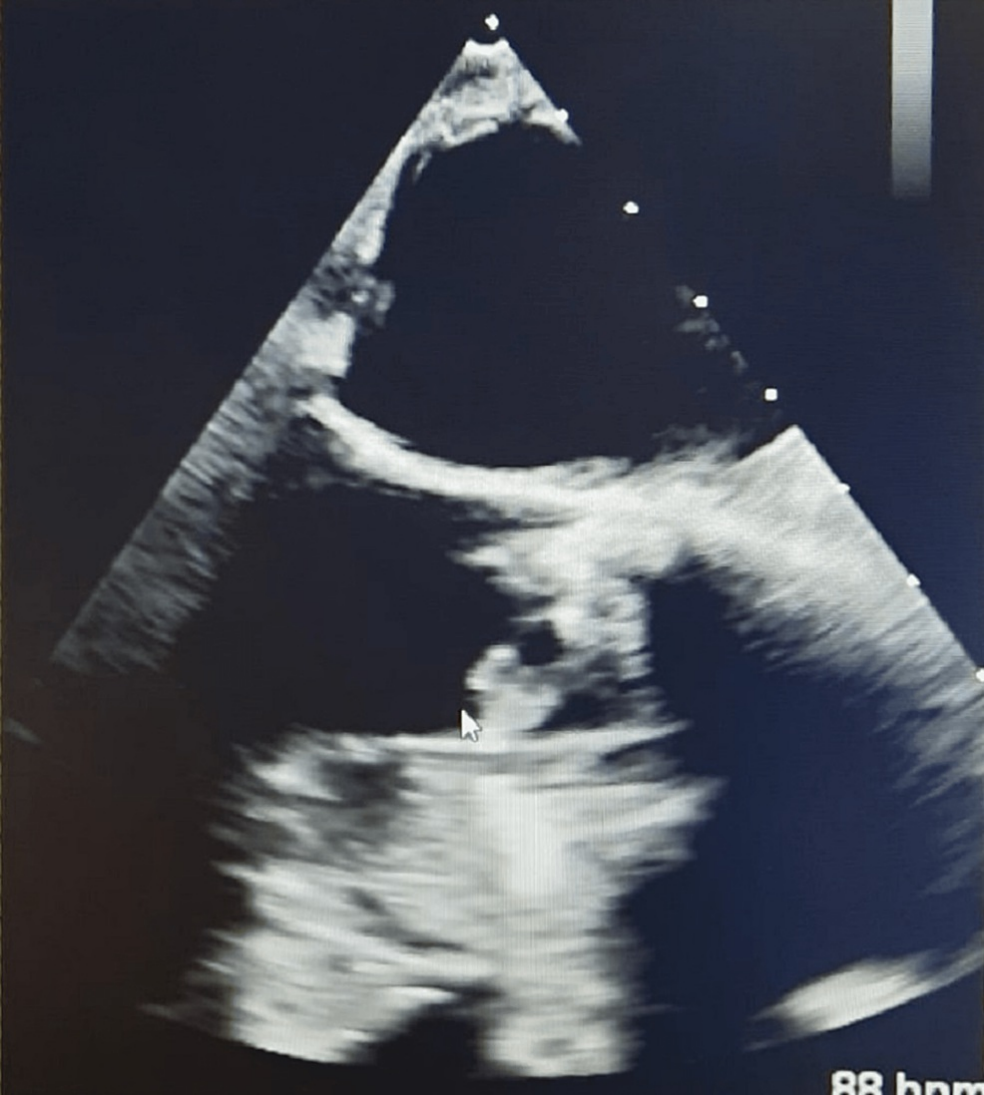
Mobile multilobed mass on the LVOT surface of an aortic prosthesis, representing vegetation.

**Figure 3:**
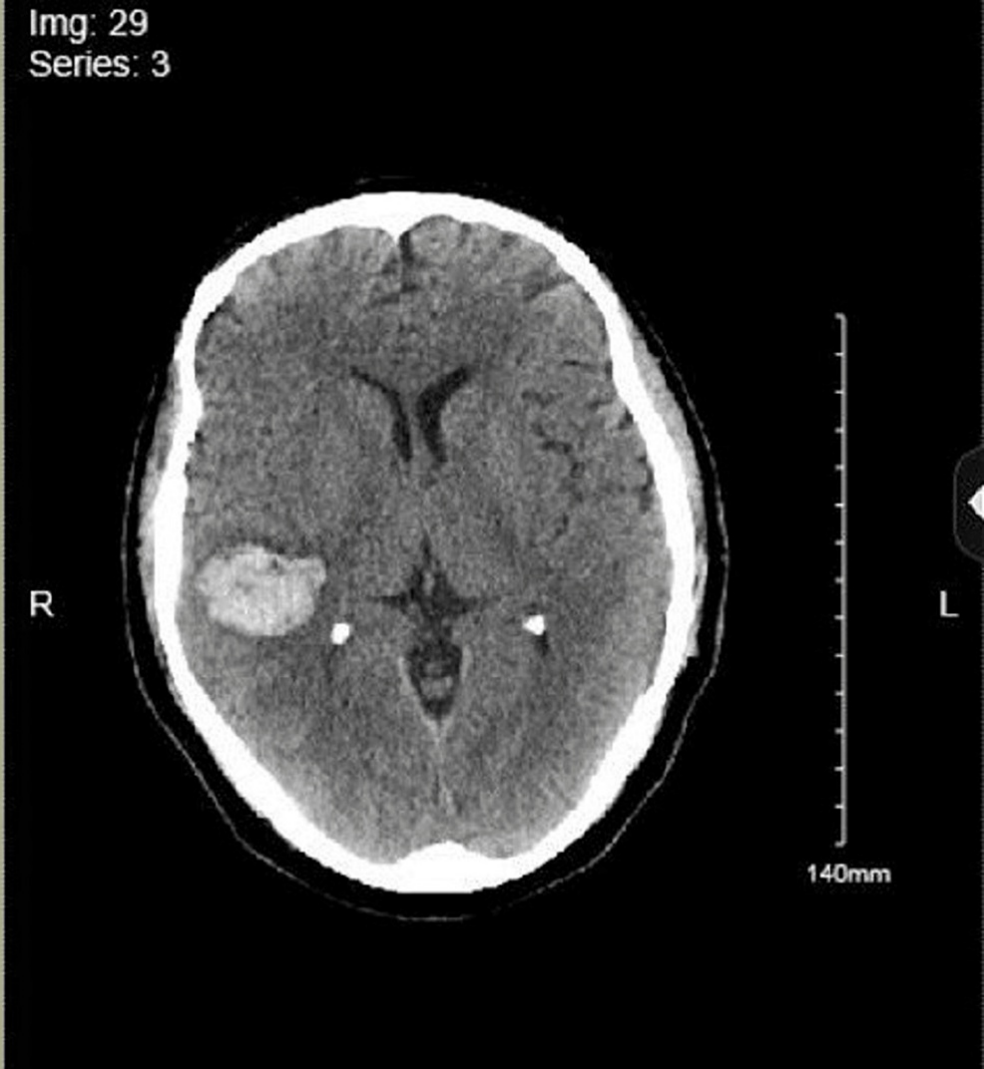
Acute frontoparietal intracranial hemorrhage.

A 59-year-old male with a past medical history of TAVR (transcatheter aortic valve replacement) for aortic regurgitation in 1995 on warfarin for anticoagulation presented to the emergency room (ER) with dull right flank pain of one day duration. The pain was non-radiating, 6/10 in severity, and without any associated symptoms, aggravating, or relieving factors. The physical examination was notable for abdominal tenderness, more prominent in the right lumbar region. Abdominal examination was otherwise benign, with a lack of rebound tenderness and normal bowel sounds. He was also noted to have poor dentition on examination. Vitals in the ER were notable for a blood pressure of 128/62 mmHg, a pulse rate of 108/min, a respiratory rate of 18/min, a temperature of 101.5 F, and saturating 100% on room air (pulse oximetry). Labs revealed an elevated white cell count of 12,500/microliter. Warfarin was held after the international normalized ratio (INR) was noted to be elevated at 11. Of note, there was no prior history of elevated INR readings, and the elevated INR was likely from his additional dose of warfarin that he took by mistake two days prior. Other labs, including vitamin K (0.4 ng/dL), activated partial thromboplastin time (aPTT) (42 seconds), D-dimer (< 0.5), and fibrinogen (270 mg/dL), were normal, including his liver function tests. Creatinine was elevated to 1.6 mg/dL (the patient had no history of underlying kidney disease; baseline creatinine was 0.8-0.9 mg/dL). A CT abdomen with contrast revealed the presence of peripheral wedge-shaped lesions in the splenic and bilateral kidneys concerning splenic and renal infarcts (Figure 1). Following the activation of the sepsis bundle in the ER, the patient received intravenous fluids (30 cc/kg), blood and urine cultures were sent, and the patient was started on vancomycin and ceftriaxone. On further evaluation, TEE revealed a mobile multilobed mass on the left ventricular outflow tract (LVOT) surface of the aortic prosthesis, representing vegetation (Figure 2). Antibiotics were transitioned to cefazolin 2g/q8h, gentamycin 3 mg/kg/q24h, and Rifampin 300mg/q8h for the management of prosthetic valve endocarditis and given positive blood cultures for staph epidermidis. The patient's INR improved to 3.5 on the third day of hospitalization, and heparin was initiated to maintain anticoagulation for the prosthetic valve. However, on the eighth day of hospitalization, the patient developed acute frontoparietal intracranial hemorrhage (ICH) with an INR elevated at 5 and an aPTT of 80.4 seconds (Figure 3). The National Institutes of Health stroke scale (NIHSS) score was 6. Heparin was reversed with protamine sulfate, and vitamin K was administered, following which the INR improved to 2.3. The patient was transferred to intensive care, but on the second day of the ICU stay, the INR again shot up to 6. The patient received vitamin K, which helped lower the INR, but was only able to improve it to 5. He underwent workup for correlation factors, which revealed decreased Factor VII (17%, normal: 50%-200%), Factor IX (44, normal: 50%-150%), Factor X (42 %, normal: 76%-183%), and normal activity with the rest of the factors. Subsequently, antibiotics were changed from cefazolin to nafcillin due to concern for cefazolin-induced coagulopathy. INR consequently fell to 1.6 in two days after changing the antibiotics. The patient was soon transferred to a higher center for aortic valve replacement. 

## Discussion

An elevated international normalized ratio after the use of cephalosporin is a complication that has been documented a few times in the literature. Cefazolin, a first-generation antibiotic, and Ceftaroline, a fifth-generation antibiotic, are the common culprits that have been documented in scholarly articles. According to an article by Barnes et al., a similar case of elevated international normalized ratio (INR) was reported after cefazolin was used to treat osteomyelitis in a COVID-19 patient. This patient was also on apixaban and was later found to have bleeding in the internal jugular vein catheter [6]. Furthermore, in another case report by Almeida et al., cefazolin was used in combination with rifampin for the treatment of septic arthritis and was noted to have caused elevated bleeding at the surgical site post-debridement [7]. 

Although the main mechanism of elevated INR is not well understood, a few theories have been propounded. Firstly, cephalosporins may eradicate intestinal flora, thereby reducing the amount of vitamin K, leading to elevated INR. Another mechanism that has been well documented is that cephalosporin itself reduces the production of vitamin K in the liver. The biochemical structure of the cephalosporins that have been implicated in this phenomenon also plays a major role. Cefazolin and Ceftaroline have been noted to have a thiadiazole side chain. The cephalosporins without this side chain didn’t exhibit the complications of elevated INR. Therefore, this side chain could be another reason why there are increased bleeding tendencies when taking this group of cephalosporins [8]. Patient factors also play an important role in the pathological process. Comorbidities such as nutritional deficiencies, renal failure (acute and chronic), and hypoalbuminemia have been noted to increase the risk of bleeding tendencies. Nutritional deficiencies lead to a decreased amount of oral intake of vitamin K, and renal failure leads to decreased clearance of the medication, thereby potentiating its toxicity. Another important factor to note is the combination of cephalosporin with another medication, which can increase the risk of bleeding. A good example of such medication is rifampin. The patient in the article by Barnes et al. had malnutrition, and the patient in the article by Almeida et al. had stage 3 chronic kidney disease, and this goes on to buttress the point made about patient comorbidities. Our patient was also on a combination of cefazolin and rifampin, similar to the patient in the article by Almeida et al. The bleeding tendencies may present as early as less than a week or later, up to more than a month after the initiation of cephalosporins. The bleeding episodes may present in different ways, from mild (bruising, ecchymoses) to severe (gastrointestinal bleeding, intracranial bleeding, hemarthrosis). In most cases, switching to a different antibiotic coupled with the administration of Vitamin K almost always leads to INR becoming normalized.

In summary, elevated INR as a complication of cephalosporins is less frequently encountered; however, there is always a need to look out for it, especially in patients with the above-mentioned risk factors.

## Conclusions

Patients on cefazolin need to be closely monitored for INR levels every day, particularly in the high-risk group, as there is a high likelihood of developing complications like ICH, as observed in this patient. Although this effect is secondary to cefazolin itself, it's not completely understood if it is dose-dependent. While monitoring the levels of cefazolin is not necessarily indicated, it is necessary to place the patients on fall precautions and monitor INR levels every day, as mentioned above.
